# Evaluation of anthocyanins in *Aronia melanocarpa*/BSA binding by spectroscopic studies

**DOI:** 10.1186/s13568-018-0604-5

**Published:** 2018-05-02

**Authors:** Jie Wei, Dexin Xu, Xiao Zhang, Jing Yang, Qiuyu Wang

**Affiliations:** School of Life Science of Liaoning University, Chongshan Middle road 66, Huanggu District, Shenyang, 110036 Liaoning China

**Keywords:** Anthocyanins in *Aronia melanocarpa*, BSA, Binding mode, Circular dichroism, Molecular docking

## Abstract

The interaction between Anthocyanins in *Aronia melanocarpa* (AMA) and bovine serum albumin (BSA) were studied in this paper by multispectral technology, such as fluorescence quenching titration, circular dichroism (CD) spectroscopy and Fourier transform infrared spectroscopy (FTIR). The results of the fluorescence titration revealed that AMA could strongly quench the intrinsic fluorescence of BSA by static quenching. The apparent binding constants K_SV_ and number of binding sites n of AMA with BSA were obtained by fluorescence quenching method. The thermodynamic parameters, enthalpy change (ΔH) and entropy change (ΔS), were calculated to be 18.45 kJ mol^−1^ > 0 and 149.72 J mol^−1^ K^−1^ > 0, respectively, which indicated that the interaction of AMA with BSA was driven mainly by hydrophobic forces. The binding process was a spontaneous process of Gibbs free energy change. Based on Förster’s non-radiative energy transfer theory, the distance r between the donor (BSA) and the receptor (AMA) was calculated to be 3.88 nm. Their conformations were analyzed using infrared spectroscopy and CD. The results of multispectral technology showed that the binding of AMA to BSA induced the conformational change of BSA.

## Introduction

**Scheme 1:**
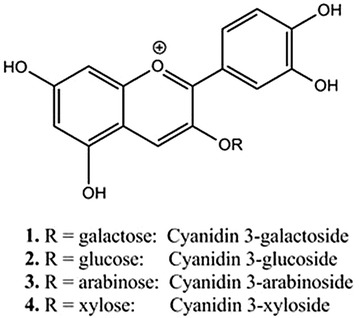
The structure of anthocyanins

*Aronia melanocarpa* Elliot, a member of the *Rosaceae* family, *Aronia melanocarpa* fruits are one of the richest plant sources of anthocyanins, AMA are water-soluble plant pigments, it has gained popularity due to their high content anthocyanins with antioxidant anti-inflammatory, antimicrobial, hepatoprotective, gastroprotective and other activities (Malinowska et al. 2013; Fares et al. 2011; Kokotkiewicz et al. 2010; Chrubasik et al. 2010). AMA have the better abilities on scavenging free radicals, improving immunity, anti-cancer, anti-aging, anti-cardiovascular disease and so on (Wei et al. 2017, 2016). The basic structure of AMA shown in Scheme 1, the main components of its monomer are cyanidin-3-*O*-arabinoside, cyanidin-3-*O*-galactoside, cyanidin-3-*O*-glucoside and cyanidin-3-*O*-Xyloside. In our previous study, we have carried out a series of optimization on the extraction and purification of AMA, its composition and biological activity were initially identified and studied (de Santiago et al. 2014). Based on this study, it was found that AMA can inhibit the occurrence of diabetes and obesity, and regulate the metabolism balance and the stability of the redox system, we also carried out AMA on mouse aging mechanism of intervention. Research also shows AMA can be used as food additives owing to its strong antioxidant capacity (Hassellund et al. 2012).

Bovine serum albumin (BSA), one of the major components in plasma protein, is the most extensively studied serum albumin, due to its structural homology with HAS(Manikandamathavan et al. 2017). we investigated the binding and associated energy transfer effects of AMA with BSA. A model of this interaction is proposed in which the intrinsic fluorescence of BSA has been quenched by AMA binding by a static quenching procedure. It was found that the hydrophobic interaction between AMA and BSA played a major role in combination of thermodynamic parameters. FTIR and CD analysis showed that AMA significantly affected the polarity and hydrophobicity of tyrosine and tryptophan in BSA, which could influence the composition of BSA secondary structure, alter the conformation of protein, and further confirm the interaction between AMA and BSA.

The binding of AMA to BSA can alter the pharmacology and pharmacodynamics of these compounds such as their distribution. Therefore, the study of interaction between AMA and BSA binding through spectroscopic techniques is necessary, It laid the foundation for the study of the stability of AMA and BSA (Zhang et al. 2008a, b; Zhang et al. 2012; Sedighipoor et al. 2017).

## Materials and methods

Bovine serum albumin (BSA) was purchased from Xi’an Rui Xi Biological Technology Co., Ltd; *Aronia melanocarpa* Elliot fruit was provided by Liaoning Academy of Forestry (Shenyang, China); Anthocyanin standards (cyanidin-3-*O*-arabinoside, cyanidin-3-*O*-galactoside, cyanidin-3-*O*-glucoside and cyanidin-3-*O*-Xyloside) were purchased from Weikeqi Biotechnology Co., Ltd.

### Fluorescence quenching titration

5.0 mL anthocyanins solution (1.0 μM) was titrated by successive additions of BSA solution with the concentration of 1.0 × 10^−5^ mol L^−1^ at different conditions (T = 297, 317, 337 K). The fluorescence quenching of Bovine serum albumin (BSA) with the addition of AR1/AG 50 was recorded in the range of 290–450 nm by fluorescence spectrofluorimeter. The width of the excitation and emission slit was adjusted at 5 nm, and the excitation wavelength was selected at 280 nm. The temperature of samples was kept by recycle water during the whole experiment. All fluorescence titration experiments were done manually by 100 mL microsyringe (Zhang et al. 2013).

### Fourier transform infrared spectroscopy (FTIR) analysis

FTIR spectra of AMA and BSA were recorded on Nicolet-6700. FTIR spectrometer via the attenuated total reflection (ATR) at a resolution of 4 cm^−1^ and 32 scans in the range of 400–4000 cm^−1^ at room temperature. The corresponding absorbance contributions of BSA and Anthocyanins solutions were recorded and digitally subtracted with the same instrumental parameters, and their FTIR spectra was done by OMNIC (Li et al. 2016).

### Circular dichroism (CD) studies

The optical chamber of the CD spectrometer was deoxygenated with dry nitrogen before used and kept in a nitrogen atmosphere during experiments. The scanning speed was 60 nm min^−1^, the spectral resolution was 0.2 nm, the response time was 0.25 s, and the slit width was 1 nm. The samples were scanned at 190–250 nm. The composition and content of the secondary structure of the protein were fitted using the Origin program, and the CDpro software was used to fit the protein (BSA) secondary structure.

### Molecular docking studies

Molecular docking were carried out to visualize the binding site of AMA to BSA. All the docking calculations were performed by using Autodock 4.2.1.5 Tools (Molecular Graphics Laboratory, The Scripps Research Institute). The 3D structure of four anthocyanins was downloaded from PUBCHEM-OPEN CHEMISTRY DATABASE (https://pubchem.ncbi.nlm.nih.gov/substance). Both BSA and four anthocyanins molecules were prepared using AutoDockTools 1.5.6 before docking, The docking was carried out with 126 × 126 × 126 0.375 Å spacing grids covering the entire surface of BSA. The Lamarckian genetic algorithm, which is considered one of the most appropriate docking methods available in AutoDock, was used in the docking analysis (Paul et al. 2017).

### Molecular dynamic (MD) simulations

The results of Molecular docking simulations determine a general binding mode of ligand. Nevertheless, MD simulation on the ligand–protein complex for further investigation of the effects of ligand binding on the conformation of protein was used. A MD simulation was performed using the AutoDockTools-1.5.6 software package. The crystal structure of BSA complex was downloaded from the Protein Data Bank (RCSB). The model of four anthocyanins monomer were constructed using the Chem3D 16.0 software package (Zhang et al. 2015).

## Results

### Fluorescence spectra of interaction between different anthocyanins in *Aronia melanocarpa* and BSA

**Fig. 1:**
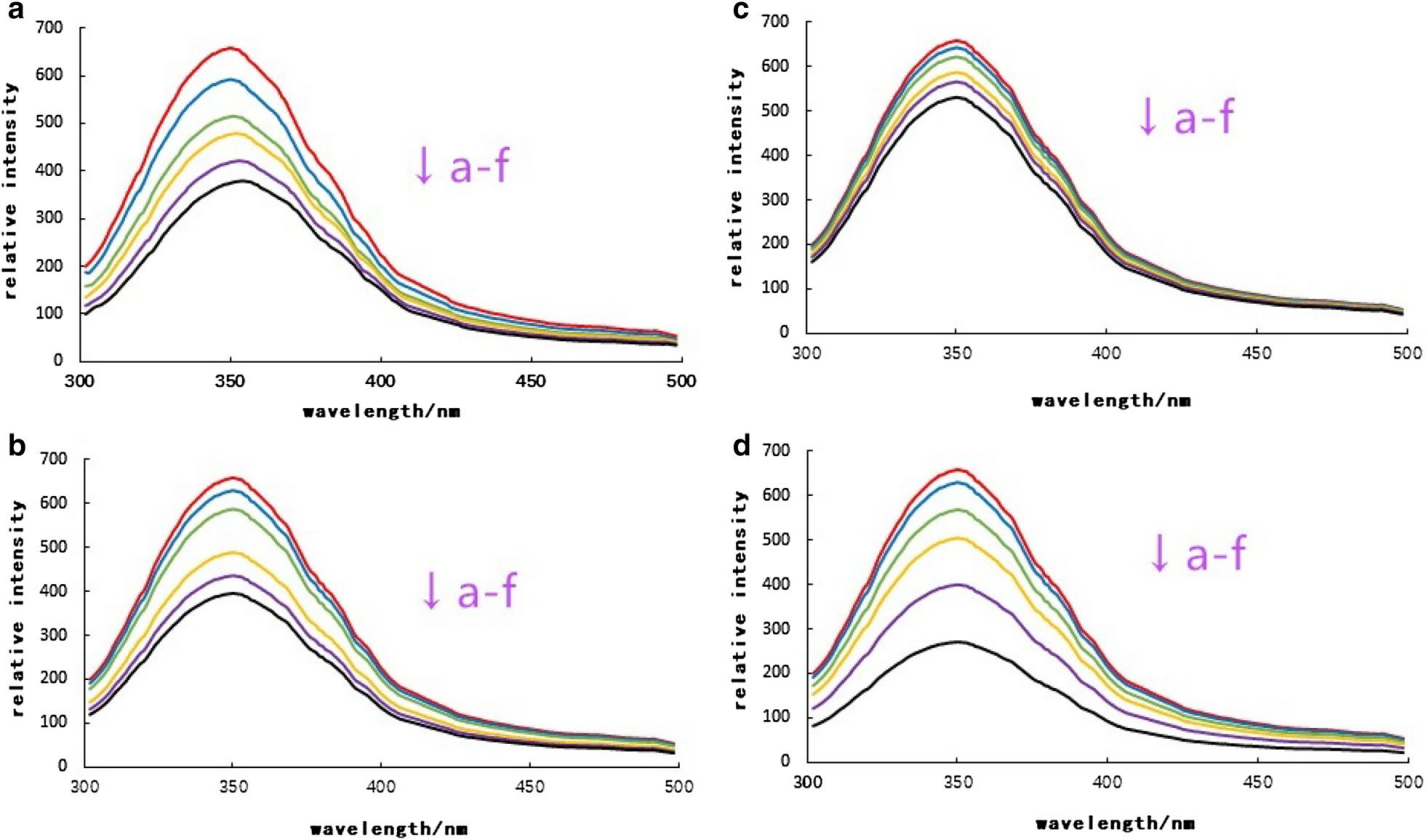
Fluorescence emission spectra of BSA suspension at excitation wavelength 280 nm in presence of 0, 5, 10, 20, 30, 40 μmol/L (a–f) Cyanidin-3-*O*-arabinoside (**a**), Cyanidin-3-*O*-galactoside (**b**), Cyanidin-3-*O*-glucoside (**c**) and Cyanidin-3-*O*-Xyloside (**d**)

Qualitative analysis of binding of AMA to BSA can be detected by examining fluorescence spectra. Generally, the fluorescence of protein is caused by three intrinsic fluors present in the protein, such as tryptophan, tyrosine, and phenylalanine residues. The fluorescence quenching pattern of BSA was shown in Fig. 1. The figure showed the fluorescence spectrum of the protein when the excitation wavelength is 280 nm, the maximum fluorescence emission wavelength (λ_max_) of BSA is about 330 nm. The fluorescence intensity at λ_max_ decreases with the increase of anthocyanin concentration, and λ_max_ has red shift phenomenon, which indicates that the microenvironment near the tryptophan and tyrosine residues in this protein was enhanced and the hydrophobicity was decreased. With the increase of the concentration of arabinoside and glucoside, the λ_max_ of BSA appeared blue shift, indicating that the polarity of the binding cavity near the tryptophan residue was weakened and the secondary structure changed (Gallo et al. 2013). In Fig. 1, the λ_max_ of BSA did not change significantly, indicating that the microenvironment of the tryptophan residue did not change. According to the fluorescence data of λ_ex_ = 280 nm, the quenching rates of cyanidin-3-*O*-arabinoside, cyanidin-3-*O*-galactoside, cyanidin-3-*O*-glucoside and cyanidin-3-*O*-Xyloside were 25, 31, 30, 32%. Different quenching rates may be related to the reaction process, the results showed that the quenching rate: The extent of reaction cyanidin-3-i-Xyloside and BSA was the most obvious (Zhang et al. 2008a, b; Unnikrishnan et al. 2014).

### Quenching mechanism of BSA fluorescence by AMA

**Fig. 2:**
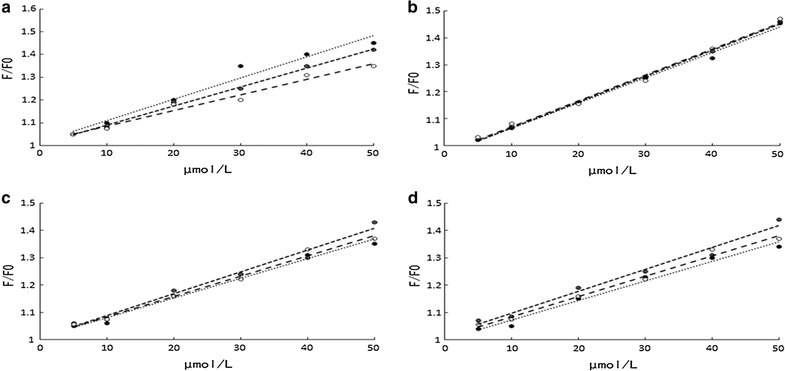
The Stern–Volmer curves of Cyanidin-3-*O*-arabinoside (**a**), Cyanidin-3-*O*-galactoside (**b**), Cyanidin-3-*O*-glucoside (**c**) and Cyanidin-3-*O*-Xyloside (**d**) at 297, 317 and 337 K

From Fig. 2 it is clear that fluorescence of BSA has been completely quenched by Anthocyanins. The quenching constants has been calculated according to the Stern–Volmer equation Eq. (1),

1 $$ {\text{F}}_{0} /{\text{F}} = 1 + {\text{K}}_{\text{SV}} \cdot {\text{C}}_{\text{q}} = 1 - {\text{K}}_{\text{q}}\uptau_{0} {\text{C}}_{\text{q}} $$

wherein, F and F_0_ are the fluorescence intensity before and after the action of the fluorescence quencher molecule, K_SV_ is the Stern–Volmer dynamic quenching constant, C_q_ is the quencher concentration, K_q_ is the rate constant of the biological macromolecule quenching process, τ_0_ is the lifetime of the fluorescent molecules (10^−8^ s) when the quencher is absent. According to the formula, the Stern–Volmer (S-V) curve of BSA interacting with four monomer can be obtained by plotting F_0_/F to C_q_. The S-V curve of the protein was in a non-linear relationship, indicating that the quenching of the BSA endogenous fluorescence was not caused by dynamic quenching, possibly due to the formation of non-luminescent complexes between the fluorescent molecules and the quenchers things (Soares et al. 2007).

### Dynamic quenching constants of BSA at different temperatures

The dynamic quenching constant K_SV_ of the protein at different temperatures (297, 317 and 337 K) and the dynamic quenching process rate constant Kq were shown in the Table 1. The results showed that when the fluorescence quenching mechanism of the protein was dynamic quenching, K_SV_ generally increased with the temperature of the system, and the maximum diffusion collision quenching constant of the quencher to the biological macromolecule was about 2 × 10^10^ L Mol^−1^ s^−1^. It could be seen that the value of K_SV_ decreased with the increase of temperature, and the fluorescence quenching rate constants of the four anthocyanins were much larger than 2 × 10^10^ L mol^−1^ s^−1^. It was shown that the quenching mechanism of the four monomer was not a dynamic quenching caused by diffusion and collision, but because of the static quenching caused by the formation of non-luminescent ground state complexes between the fluorescent molecules and the quencher (Sun et al. 2017).

**Table 1:** The dynamic quenching constants of Cyanidin-3-*O*-arabinoside, Cyanidin-3-*O*-galactoside, Cyanidin-3-*O*-glucoside and Cyanidin-3-*O*-Xyloside at 297, 317 and 337 K

Complexes	T/K	K_SV_/(× 10^3^ L mol^−1^)	K_q_/(× 10^11^ L mol^−1^)
Cyanidin	297	10.227 ± 0.003	10.227 ± 0.003
-3-*O*-	317	9.984 ± 0.156	9.984 ± 0.156
Arabinoside	337	9.166 ± 0.197	9.166 ± 0.197
Cyanidin	297	8.795 ± 0.284	8.795 ± 0.284
-3-*O*-	317	8.699 ± 0.105	8.699 ± 0.105
Galactoside	337	8.012 ± 0.015	8.012 ± 0.015
Cyanidin	297	7.843 ± 0.019	7.843 ± 0.019
-3-*O*-	317	7.435 ± 0.201	7.435 ± 0.201
Glucoside	337	6.915 ± 0.174	6.915 ± 0.174
Cyanidin	297	7.065 ± 0.162	7.065 ± 0.162
-3-*O*-	317	6.845 ± 0.153	6.845 ± 0.153
Xyloside	337	6.254 ± 0.074	6.254 ± 0.074

### Determination of Binding constants, the number of binding sites and the type of binding

**Fig. 3:**
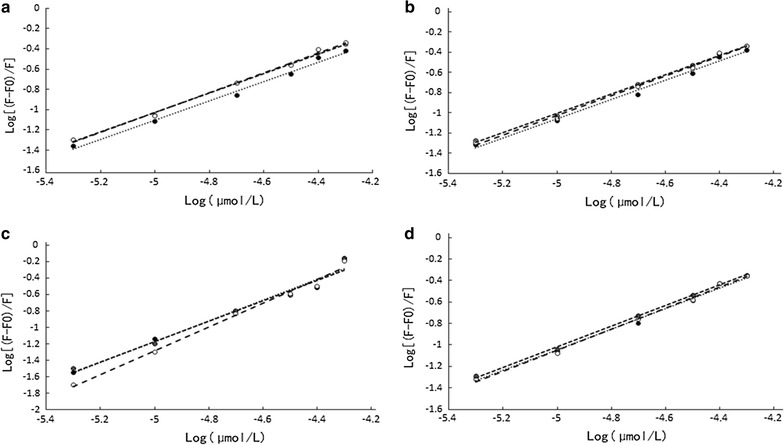
The double logarithm regression curve of log [(F_0 _− F)/F] versus log [c_q_] of Cyanidin-3-*O*-arabinoside (**a**), Cyanidin-3-*O*-galactoside (**b**), Cyanidin-3-i-glucoside (**c**) and Cyanidin-3-*O*-Xyloside (**d**) at 297, 317 and 337 K

Double logarithmic regression curves of the interaction of four anthocyanins with BSA was shown in Fig. 3 When small molecules bind independently to a set of equivalent sites on a macromolecule, the equilibrium between free and bound molecules is given by the equation Eq. (2).

2 $$ \log ({\text{F}}_{0} - {\text{F}})/{\text{F}} = \log K_{\text{s}} + {\text{n}}\log {\text{C}}_{\text{q}} $$

where K_S_ and n are the apparent binding constant and the number of binding sites. Thus, a plot (Fig. 3) of log (F_0 _− F)/F versus log(Q) yielded the K_S_ and n values to be 0.574 × 10^3^ L mol^−1^, 0.484 × 10^3^ L mol^−1^, 0.425 × 10^3^ L mol^−1^, 0.521 × 10^3^ L mol^−1^ and 0.9395, 0.9195, 0.9153, 0.9265 at 297 K as shown in Table 2, respectively. An n value of approximately equal to 1 indicated that there was only a single binding site in the binding of AMA and BSA.

**Table 2:** The binding constants and thermodynamic parameters of Cyanidin-3-*O*-arabinoside, Cyanidin-3-*O*-galactoside, Cyanidin-3-*O*-glucoside and Cyanidin-3-*O*-Xyloside at 297, 317 and 337 K

Complexes	T	K_s_ (× 10^3^ L mol^−1^)	n	∆H (KJ mol^−1^)	∆G (KJ mol^−1^)	∆S (J mol^−1^· K)
Cyanidin	297	0.574 ± 0.023	0.9395 ± 0.0034	4.03	− 22.39	88.95623
-3-*O*-	317	0.412 ± 0.037	0.9754 ± 0.0044	4.03	− 24.59	90.28391
Arabinoside	337	0.398 ± 0.010	0.9489 ± 0.0058	4.03	− 25.98	89.05045
Cyanidin	297	0.484 ± 0.025	0.9195 ± 0.0054	20.47	− 21.63	86.39731
-3-*O*-	317	0.395 ± 0.011	0.9515 ± 0.0037	20.47	− 20.84	78.45426
Galactoside	337	0.308 ± 0.012	0.9354 ± 0.0014	20.47	− 25.14	86.55786
Cyanidin	297	0.425 ± 0.037	0.9153 ± 0.0022	11.56	− 24.22	95.11785
-3-*O*-	317	0.403 ± 0.033	0.9175 ± 0.0009	11.56	− 29.23	104.9211
Glucoside	337	0.399 ± 0.030	0.9612 ± 0.0074	11.56	− 21.78	76.58754
Cyanidin	297	0.521 ± 0.027	0.9265 ± 0.0085	9.81	− 28.47	109.4276
-3-*O*-	317	0.543 ± 0.026	0.8987 ± 0.0045	9.81	− 23.96	88.29653
Xyloside	337	0.537 ± 0.014	0.9024 ± 0.0049	9.81	− 25.51	87.65579

The thermodynamic constants of ligand-macromolecule binding can be calculated according to the Van’t Hoff equation Eq. (3)

3 $$ \Delta {\text{H}} = {\text{d}}\left( {\frac{\Delta G}{T}} \right)/{\text{d}}\left( {\frac{1}{T}} \right) $$

$$ \Delta {\text{G}} = - {\text{RT}}\;\ln {\text{K}}_{\text{s}} $$

$$ \Delta {\text{G}} = \Delta {\text{H}} - {\text{T}}\Delta {\text{S}} $$

wherein the K_S_ binding constants representative of the temperature T, ΔH, ΔS, ΔG, respectively enthalpy change of the bonding process, entropy and free energy, R is the gas constant (8.314 J mol^−1^ K^−1^). The values of ∆H, ∆G, and ∆S are listed in Table 2. From the point of view of water structure, a positive ∆S value was frequently taken as evidence for hydrophobic interaction. The negative value of ∆G revealed that the interaction process was spontaneous (Pomar et al. 2005). From the results we can conclude that the ΔG of the binding of the four monomers to BSA was less than 0, indicating that the reaction between the four monomers was spontaneous; ΔH(BSA) > 0, ΔS(BSA) > 0, indicating that the effect of BSA was mainly hydrophobic. ΔH(BSA) > 0, the reaction was endothermic reaction, the K_S_ value increased with increasing temperature. The interaction forces between a small molecule and macromolecule include hydrogen bonds, van der Waals force, hydrophobic force, electrostatic interactions, etc. In order to elucidate the interaction of AMA with BSA, the thermodynamic parameters were calculated.

### Fourier transform infrared spectroscopy (FTIR) analysis

**Fig. 4:**
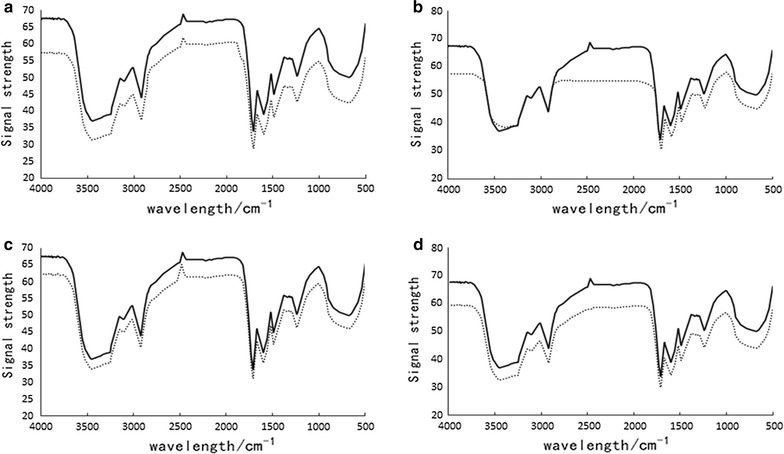
FTIR spectra in the region 4000–400 cm^−1^for Cyanidin-3-*O*-arabinoside (**a**), Cyanidin-3-*O*-galactoside (**b**), Cyanidin-3-*O*-glucoside (**c**) and Cyanidin-3-*O*-Xyloside (**d**) and their polyphenol complexes

The infrared spectrum was shown in Fig. 4, Changes in the infrared spectrum indicated that the four monomer caused a change in the secondary structure of the BSA. Several oxygen atoms, hydroxyl and BSA C=O, C–N groups through the hydrogen bond, hydrophobic interaction combined to form a complex, resulting in BSA peptide chain rearrangement, and ultimately leaded to secondary structure changes(Hu et al. 2004).

### Circular dichroism analysis

**Fig. 5:**
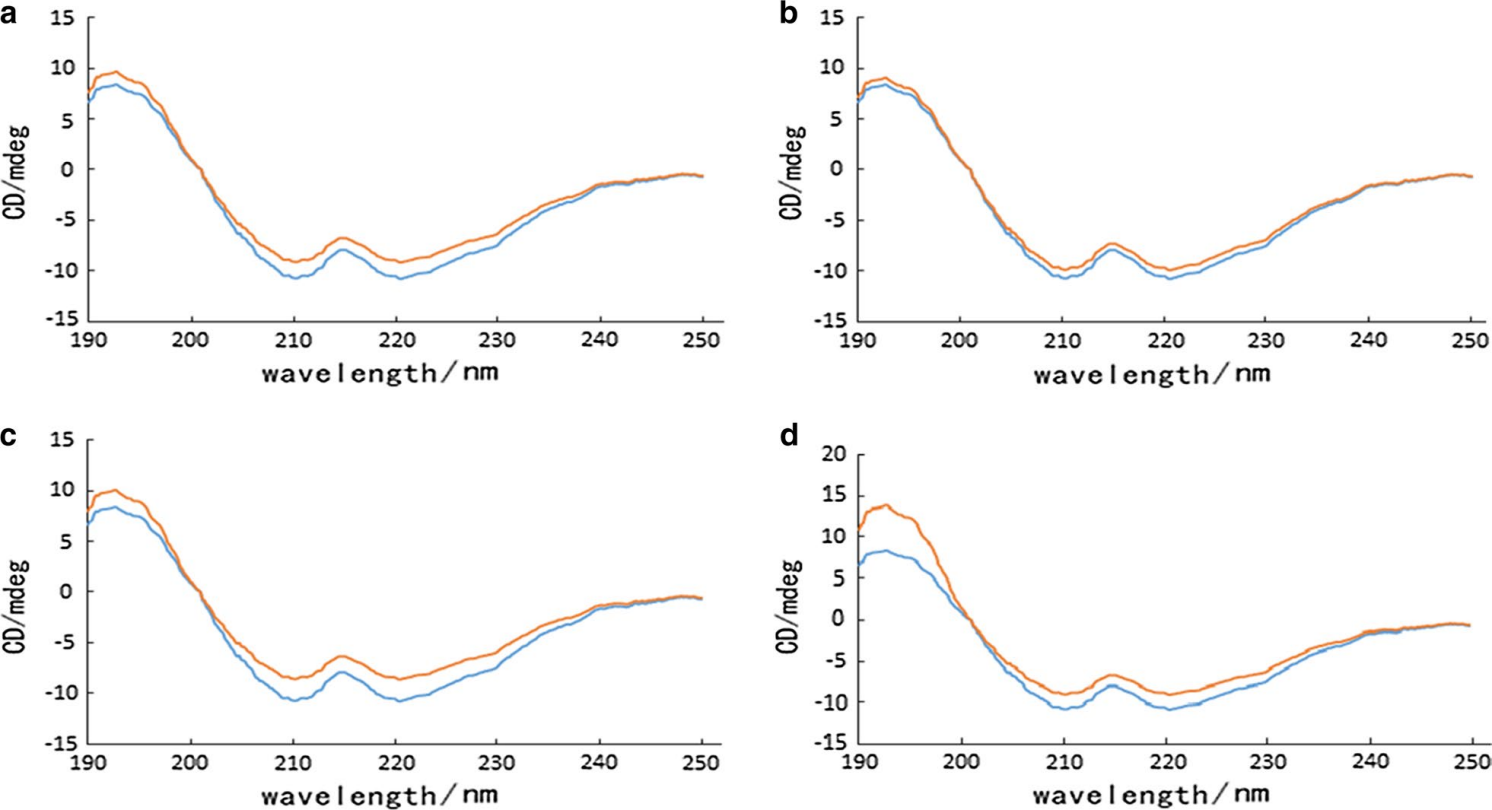
The far-UV CD spectra of Cyanidin-3-*O*-arabinoside (**a**), Cyanidin-3-*O*-galactoside (**b**), Cyanidin-3-*O*-glucoside (**c**) and Cyanidin-3-*O*-Xyloside (**d**) in absence and presence of BSA

Circular dichroic analysis is a useful method for secondary structure estimation of protein molecule. The shape and particular wavelengths of CD spectrums are very sensitive to the secondary structure of proteins. The secondary structure changes of BSA in the presence of the four monomer were studied using circular dichroism spectroscopy. Figure 5 shows CD analysis of BSA in the absence and presence of four monomer, the interaction between AMA and BSA could be verified (Wawer et al. 2006; Slimestad et al. 2005). The four monomer were listed in Table 3 by software, we can conclude that after addition of four monomer, α-helix content of BSA was almost no change, β-fold content was increased, but corner and random curl content was decreased (Karnaukhova 2007). So the CD results demonstrated that the interaction of the four monomer with the BSA leaded to a change in the secondary structure of the BSA, which was consistent with the infrared spectrum (Sahu et al. 2008).

**Table 3:** Secondary structure analysis from the BSA and AMA

AMA (10 μmol L^−1^)	α-helix (%)	β-fold (%)	β-angle (%)	Random curl (%)
BSA	10.2	9.1	33.2	47.5
With cyanidin-3-*O*-arabinoside	9.2	8.9	35.0	46.9
With cyanidin-3-*O*-galactoside	9.0	9.1	34.7	47.2
With cyanidin-3-*O*-glucoside	9.1	8.8	34.1	48.0
with cyanidin-3-*O*-Xyloside	9.2	8.9	33.9	48.0

### Computational analysis of the binding between AMA and BSA

**Fig. 6:**
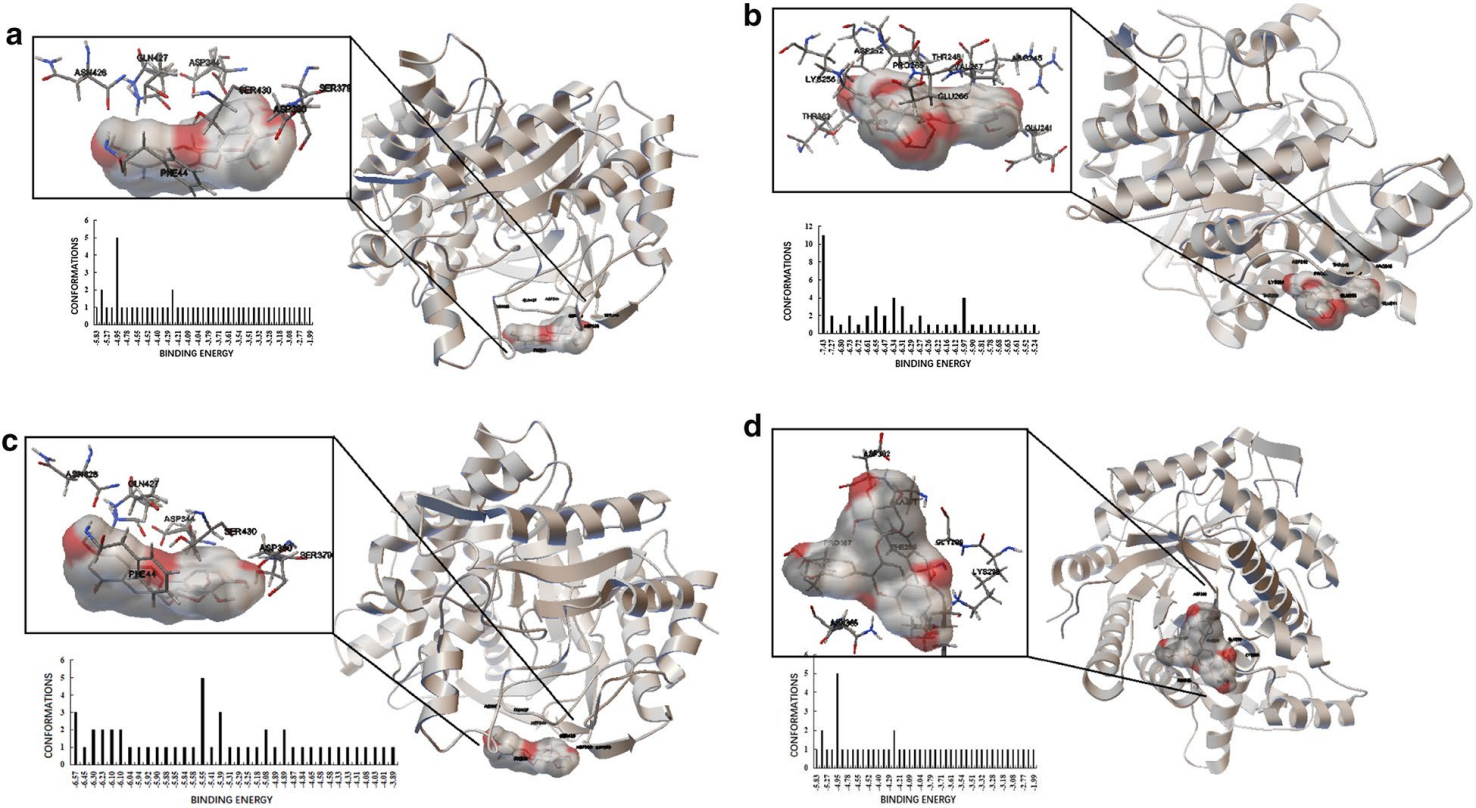
Use Autodock software binding energy of 50 docking models. Panoramic view showing the binding mode between Cyanidin-3-*O*-arabinoside (**a**), Cyanidin-3-*O*-galactoside (**b**), Cyanidin-3-*O*-glucoside (**c**) and Cyanidin-3-*O*-Xyloside (**d**), and BSA

We carried out docking simulations to investigate the possible 4 anthocyanins-binding site on BSA. The binding energy of 50 models in docking is shown in Fig. 6. In areas where these binding patterns are present, AMA may bind to BSA and is located in the region shown in Fig. 6. Consequently, the stabilizing effect contributed by AMA on the appendant structure of BSA may prevent the occurrence of domain swapping, so as to redirect BSA away from the fibril-forming pathway and into forming nontoxic, unstructured, and off-pathway aggregates (Gao et al. 2005). So we speculated that AMA could inhibit the fibrillation of BSA complex in this study. The observation was particular significance as these four anthocyanins had higher stability after interacting with BSA.

## Discussion

It is reported that the content of AMA is up to 1%, far higher than other plants (Olszewska and Michel 2009), so in this paper, the aim of above research is to clarify the binding mechanism of AMA with BSA, we will provide valuable information about interaction of AMA as a plant-based food additives with BSA as an important carrier protein, this is of great significance for the follow-up study of AMA and BSA (Li et al. 2016), and we also provide useful information for understanding the Pharmacological effects at molecular level (Zhang et al. 2012).

A series of multispectral technology and molecular docking studies, including interaction was used to analyze AMA and BSA. Fluorescence quenching showed that AMA can quench the fluorescence intensity of BSA through static mechanism making it possible to study the interaction of AMA with this protein using Stern–Volmer equation.

The results obtained from FTIR to CD showed that the α-helix content of BSA did not change significantly in the absence and presence of the four monomer, and the content of β-sheet was increased and the curvature and random curl content were decreased. The interaction of the four substance with BSA resulted the changes of BSA in the conformation and secondary structure. According to the Förster’s non-radiative energy transfer theory, the binding distance r between AMA and BSA was calculated, the result represents a static quenching, and the binding reaction is spontaneous and is largely mediated by hydrophobic forces.

Molecular docking is a key tool in structural molecular biology. The goal of ligand–protein docking is to predict the predominant binding mode(s) of a ligand with a protein of known three-dimensional structure (Morris and Lim-Wilby 2008). The obtained molecular docking results indicated that AMA can interact with BSA, without breaking the secondary structure of BSA. Conformational studies of BSA indicate that Trp212 is involved in the interfacial formation of subdomains IIA and IIIA and that the two hydrophobic cavities are the major regions where small molecule compounds bind to proteins through molecular modeling, several anthocyanins share the same binding site. In view of above this, it is of great significance to study the combination of AMA/BSA through the multi-spectral and molecular docking described.

## Abbreviations

MD: molecular dynamic simulations
AMA: anthocyanins in *Aronia melanocarpa*
BSA: bovine serum albumin
CD: circular dichroism spectroscopy
FTIR: Fourier transform infrared spectroscopy
